# Supplementary material for: Intracellularly Released Cholesterol from Polymer-Based Delivery Systems Alters Cellular Responses to Pneumolysin and Promotes Cell Survival
Source: Metabolites. 2021 Nov 30;11(12):821. doi: 10.3390/metabo11120821 (PMC8709088; doi:10.3390/metabo11120821)
Supplement: Supplementary file 1 [file metabolites-11-00821-s001.zip › metabolites-1461072-supplementary.pdf]

Supplementary Information

Intracellularly released cholesterol from polymer-based delivery systems alters cellular responses to pneumolysin and promotes cell survival.

Tobias Kammann, Jessica Hoff, Ilknur Yildirim, Blerina Shkodra, Tina Mueller, Christine Weber, Markus H. Gräler, Ulrich A. Maus, James C. Paton, Mervin Singer, Anja Traeger, Ulrich S. Schubert, Michael Bauer, Adrian T. Press

Content

Supplementary Table S1. Primer information. ... 2

Supplementary Table S2. Size of particles. ... 3

Supplementary Table S3. Cholesterol levels in Figure 2a ... 4

Figure S1. Intensity-plotted size distribution of particles formed by nanoprecipitation measured by DLS. ... 5

Figure S2. Intensity-plotted size distribution of particles formed by microprecipitation measured by DLS. ... 6

Figure S3: PitStop-2 dependent delivery of [E100-PLGA](Chol)$_{NP}$ in HepG2 cells. ... 7

Detailed discussion of the polymer synthesis and characterization data ... 8

Figure S4: SEC elugrams (eluent THF, RI detection) of PLA-Chol-a and PLA-Chol-b. ... 9

Figure S5: ^{1}H NMR spectrum (300 MHz, $CDCl_3$) of PLA-Chol-a and assignment of the signal to the schematic representation of the structure. ... 9

Figure S6: ^{1}H NMR spectrum (300 MHz, $CDCl_3$) of PLA-Chol-b and assignment of the signals to the schematic representation of the structure. ... 10

Figure S7: Characterization of PLA-Chol-a by mass spectrometry. ... 11

Figure S8: Characterization of PLA-Chol-b by mass spectrometry. ... 12

Materials used for synthesis of PLA-Chol ... 13

Instrumentation for characterization of PLA-Chol ... 13

Experimental section for the synthesis of PLA-Chol ... 13

Supplementary Table S1. Primer information.

Sequences of primers are displayed in 5' – 3' -direction (forwards = fwd; reverse = rev). Abbreviations: Primer melting temperature = TM; base pair(s) = bp; guanosine-cytosine content = GC (%). Δ-*6-desaturase* = (*D6D*); *β-hydroxy-β-methyl-glutaryl-CoA-reductase* = (*HMGCR*); *sterol regulatory element-binding protein-1/2* = (*SREBP-1/2*).

mRNA target	Primer $_{fwd}$ 5'-3'	Primer $_{rev}$ 5'-3'	GC (%) fwd rev	TM (°C) fwd rev	Transcipt size (bp)
D6D	CTGCTGATTGG AACTGGC	CGTGGTTGGT CTTGAACAGG	55 55	59 59	127
HMGCR	GATTTCAAAG GGTACAGAGA AAGCA	AGCAGCAGGT TTCTTGTCAG T	40 48	60 58	107
SREBP-1	AGTGGTCTGG CTGCTCAATG	ATGCCTCCAG AAGTACACGG	55 55	59 59	115
SREBP-2	AGGCAGGCTT TGAAGACGAA	GTACATCGGA ACAGGCGGAT	50 55	57 59	120

Supplementary Table S2. Size of particles.

Mean size (diameter) intensity, the z-average (z-avg), and PDI (polydispersity index) of particles prepared by nano- and microprecipitation. Data obtained by DLS after precipitation and lyophilization. n. d.: not determined.

	Polymer	Drug	Particle abbreviation	After nano- or microprecipitation			After lyophilization		
				Size intensity mean	z-avg	PDI	Size intensity mean	z-avg	PDI
Nanoprecipitation	E100/PLGA (40:60)	Chol	[E100-PLGA](Chol)$_{NP}$	290	266	0.19	270	236	0.24
		-	[E100-PLGA]$_{NP}$	210	181	0.14	190	167	0.25
	PLGA	Chol	[PLGA](Chol)$_{NP}$	150	314	0.27	190	178	0.17
		-	[PLGA]$_{NP}$	170	151	0.10	200	177	0.20
	PLA-Chol	Chol	[PLA-Chol](Chol)$_{NP}$	340	4012	> 0.5	300	1108	> 0.5
		-	[PLA-Chol]$_{NP}$	250	244	0.27	150	2381	> 0.5
Microprecipitation	E100/PLGA (40:60)	Chol	[E100-PLGA](Chol)$_{MP}$	1150	691	0.48	690	5563	> 0.5
		-	[E100-PLGA]$_{MP}$	670	550	0.19	560	1330	> 0.5
	PLGA	Chol	[PLGA](Chol)$_{MP}$	740	667	0.28	580	1065	> 0.5
		-	[PLGA]$_{MP}$	750	759	0.24	380	1839	> 0.5
	PLA-Chol	Chol	[PLA-Chol](Chol)$_{MP}$	n. d.	n. d.	n. d.	830	1340	0.43
		-	[PLA-Chol]$_{MP}$	n. d.	n. d.	n. d.	870	1480	0.28

Supplementary Table S3. Cholesterol levels in Figure 2a.

Cholesterol levels are given as mean + SD for each individual group. As controls unstimulated cells in DMEM/F12 without additives (stimulation media was used). The controls were time, batch, and passage matched HepG2 cells analyzed parallel with the stimulated samples. Differences in the cholesterol baseline observed between controls might be due to batch effects (different metabolic activities of the cells in the experiment). It is of note that the relative changes between the stimulation remained constant. SD: standard deviation, nd: not determined

**Time**	**1.5 h**		**3 h**		**6 h**		**9 h**	
**Group**	**mean**	**SD**	**mean**	**SD**	**mean**	**SD**	**mean**	**SD**
control	132,4	28,0	167,4	41,1	159,7	16,7	nd	nd
MeOH	141,9	17,7	138,9	33,3	149,5	16,7	nd	nd
MeOH-Chol	212,2	78,6	173,7	46,8	178,7	20,0	nd	nd
MCD	79,1	17,5	42,3	24,0	35,5	8,7	nd	nd
MCD-Chol	233,6	47,7	274,0	60,3	371,3	37,7	nd	nd
**Nanoparticles**								
control	352	92	344	49	480	61	289	35
[E100-PLGA](Chol)	321	47	400	53	539	117	538	82
[E100-PLGA]	283	43	313	58	410	94	298	66
[PLGA] (Chol)	284	44	381	96	489	167	nd	nd
[PLGA]	229	27	374	94	290	20	nd	nd
[PLA-Chol] (Chol)	476	65	461	64	590	154	nd	nd
[PLA-Chol]	269	23	251	45	349	60	nd	nd
**Microparticles**								
control	352	92	344	49	480	61	289	35
[E100-PLGA](Chol)	355	39	335	61	411	157	355	68
[E100-PLGA]	318	47	297	13	382	68	365	58
[PLGA] (Chol)	334	53	374	91	361	82	nd	nd
[PLGA]	292	39	352	57	421	48	nd	nd
[PLA-Chol] (Chol)	433	90	472	98	642	226	nd	nd
[PLA-Chol]	283	24	294	59	407	57	nd	nd

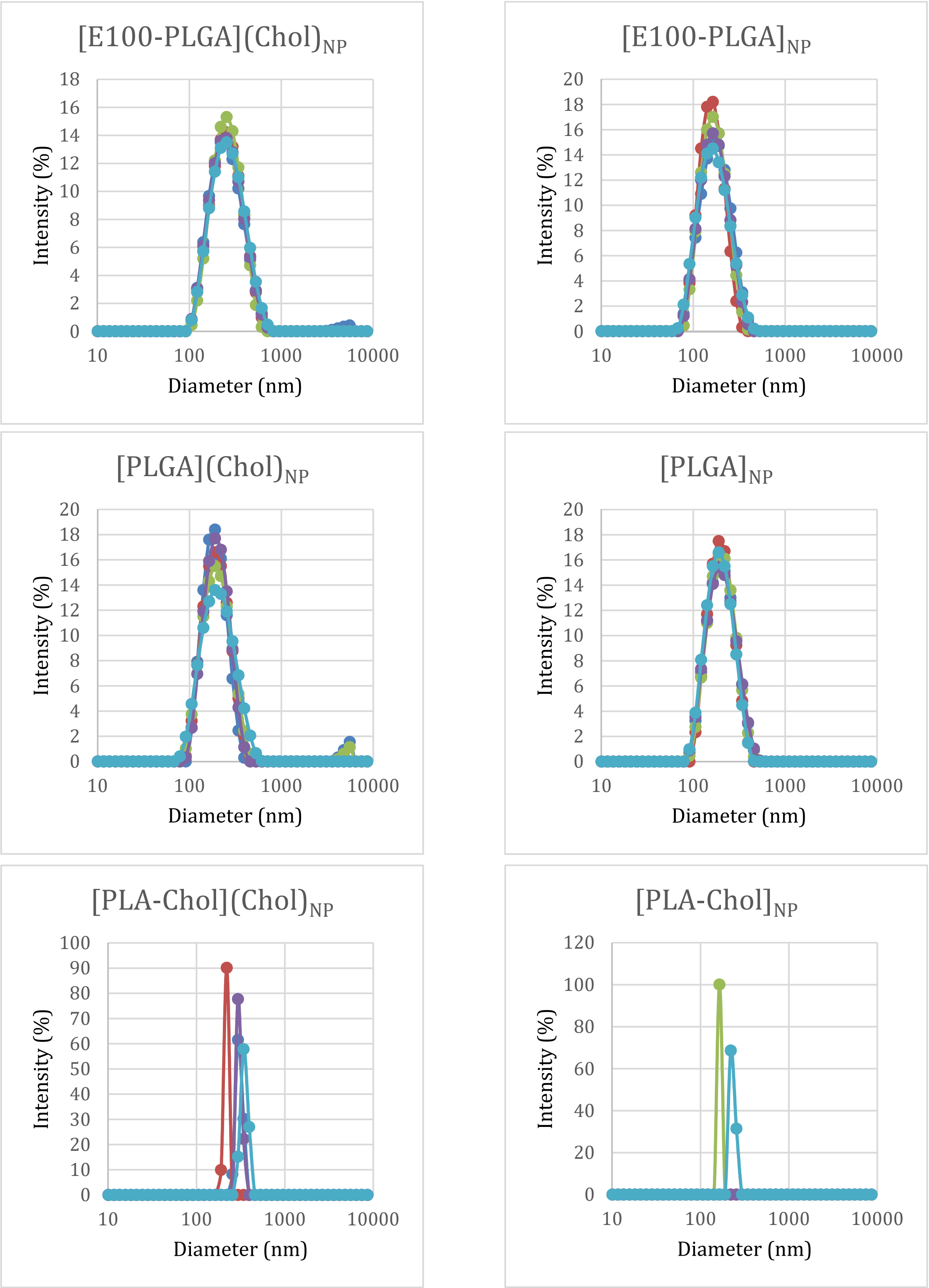

Figure S1. Intensity-plotted size distribution of particles formed by nanoprecipitation measured by DLS.

Size distribution (intensity-based) of various nanoparticles. Colors represent individual measurements.

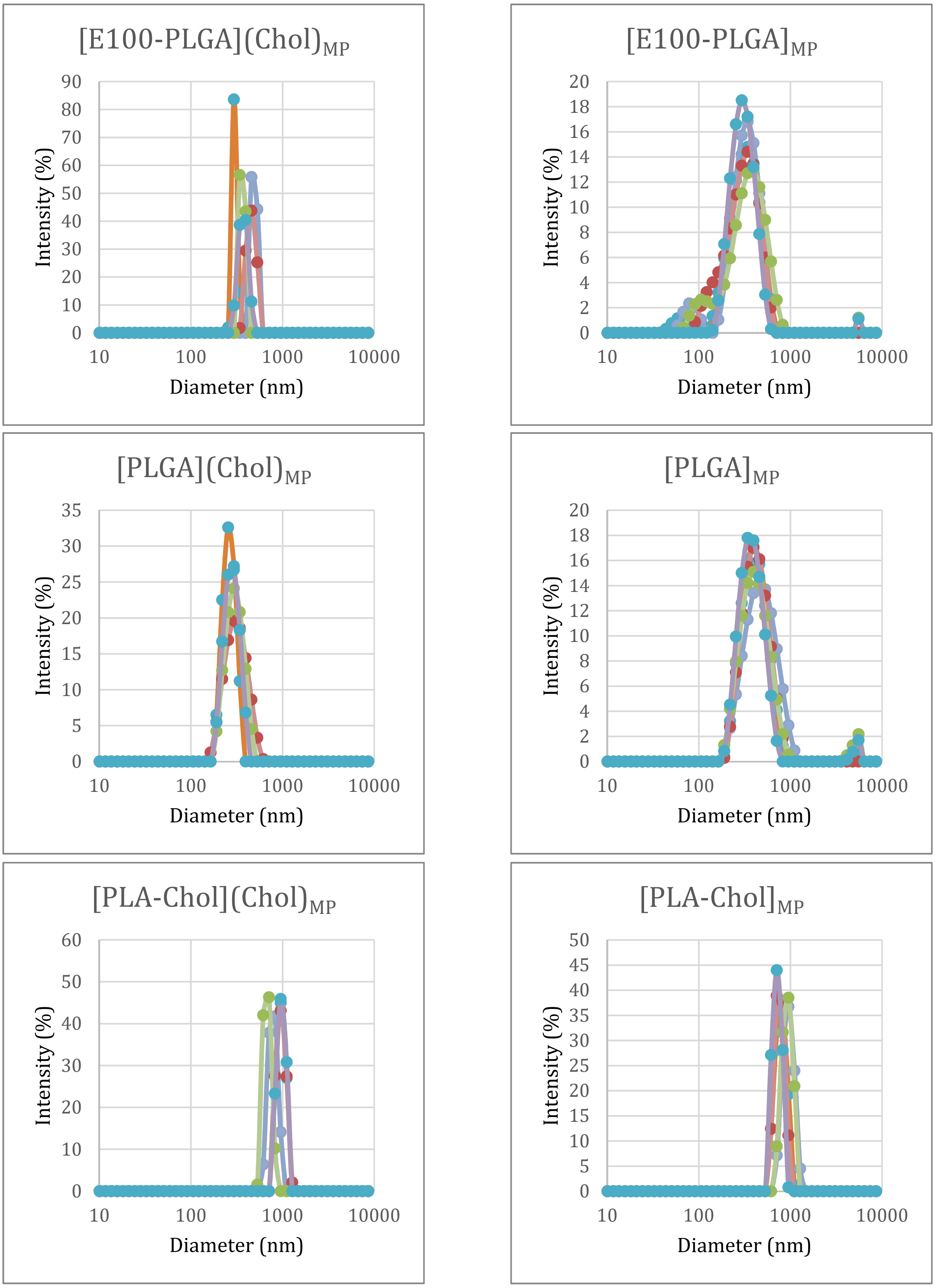

Figure S2. Intensity-plotted size distribution of particles formed by microprecipitation measured by DLS.

Size distribution (intensity-based) of various microparticles. Colors represent individual measurements.

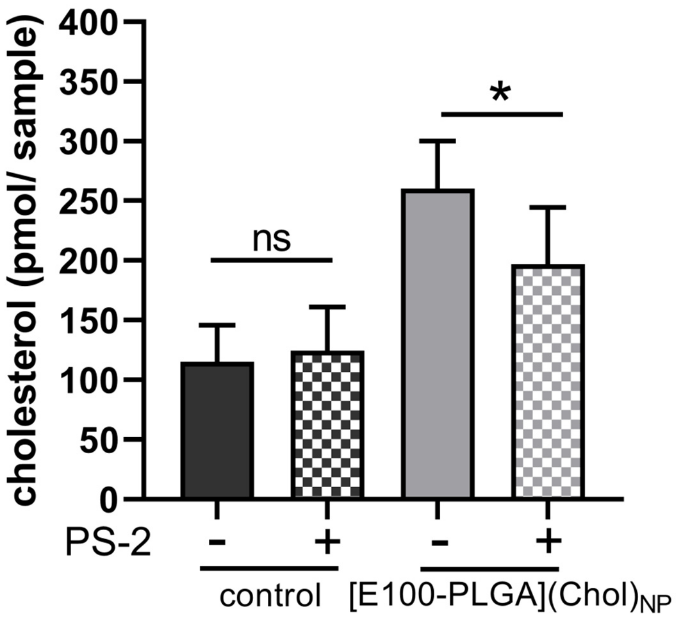

Figure S3: PitStop-2 dependent delivery of [E100-PLGA]$(Chol)_{NP}$ in HepG2 cells.

The cholesterol concentration had been analyzed by LC-MS from 45 000 cells per sample treated with PitStop-2 (PS-2) and nanoformulated [E100-PLGA]$(Chol)_{NP}$. The bar plot indicate mean + standard deviation (SD). The table specifies the absolute cholesterol concentration for each group in pmol/ sample and the number of replicates for each experiment. Statistical significance was tested between indicated groups using the Mann-Whitney Rank Sum Test (two-tailed, alpha = 0.05). ns: not significant, * $p<0.05$

A detailed discussion of the polymer synthesis and characterization data.

Alcohols are the initiators of choice for the ring-opening polymerization (ROP) of lactide to result in polylactide (PLA) with tailor-made end groups and molar masses if a suitable catalyst is chosen and the right experimental conditions for the ROP are known. Having established such conditions using the calcium-based catalyst $Ca[N(SiMe_3)_2]_2(THF)_2$ for primary, secondary as well as tertiary alcohols under mild conditions [1] that were suitable also for natural compounds such as retinol [2], the ROP of L-lactide was conducted in THF at room temperature to ensure a covalent attachment of cholesterol at the PLA carrier material (Scheme S1).

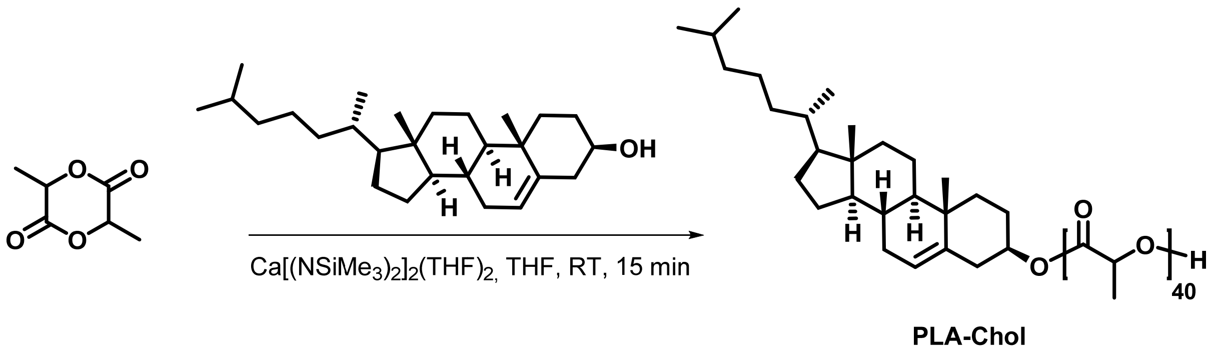

Scheme S1: Schematic representation of the ring-opening polymerization of L-lactide yielding PLA-Chol.

Because the initiating alcohol, *i. e.* cholesterol, represented the α-end group of the resulting PLA, an accurate end-group determination was inevitable. Hence, the initial ratio of [L-lactide]$_0$ / [cholesterol]$_0$ was set as 20 to produce cholesterol-functionalized PLA with a targeted molar mass of $M_{n,\ theoretical}$ = 3270 g mol^{-1}. ROP was conducted until quantitative monomer conversion after 15 min, as confirmed by ^{1}H NMR spectroscopy. SEC analysis of the reaction mixture indicated unimodal molar mass distributions with low dispersity values, not indicating the occurrence of any undesired side reactions such as intermolecular transesterification. Low molar mass fractions caused minor molar mass deviations of the purified samples (Fig. S4) during the subsequent precipitation.

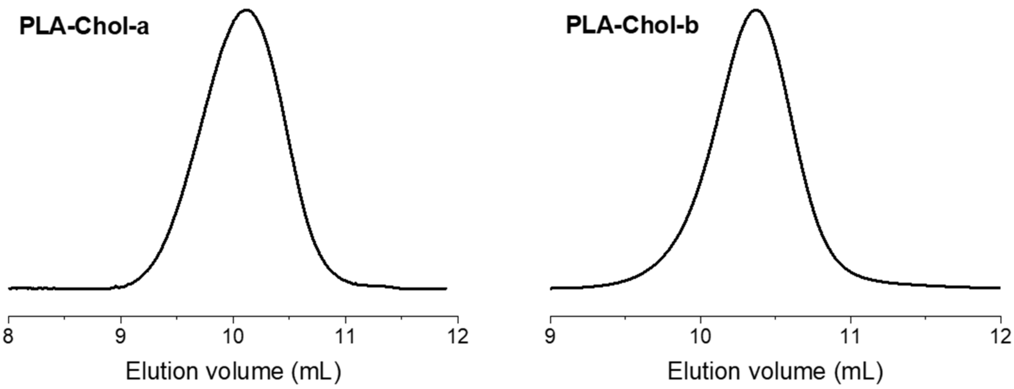

Figure S4: SEC elugrams (eluent THF, RI detection) of PLA-Chol-a and PLA-Chol-b.

More importantly, analysis of the purified PLA-Chol utilizing ^{1}H NMR spectroscopy indicated the presence of the cholesterol α-end group of the PLA (**Fig. S5**, **Fig. S6**). As several end group signals were separated from the PLA signals, the resulting molar mass of the PLA-Chol was estimated from the spectrum. The integrals of the peaks "d" assigned to the cholesterol end group and "a" given to the methine protons of the lactide repeating units were used for that purpose. The agreement of the resulting $M_{n,\ NMR}$ with the theoretically expected values and the values from SEC analysis with PLA calibration hinted towards the end group fidelity of PLA-Chol, particularly for PLA-Chol-a.

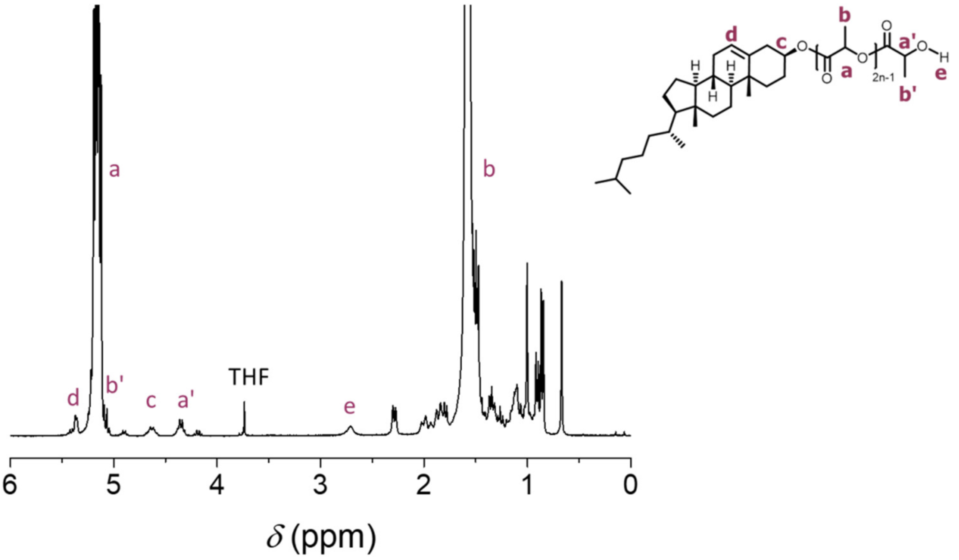

Figure S5: ^{1}H NMR spectrum (300 MHz, $CDCl_3$) of PLA-Chol-a and assignment of the signals to the schematic representation of the structure.

The peaks labeled with "a" and "d" were used to estimate the molar mass.

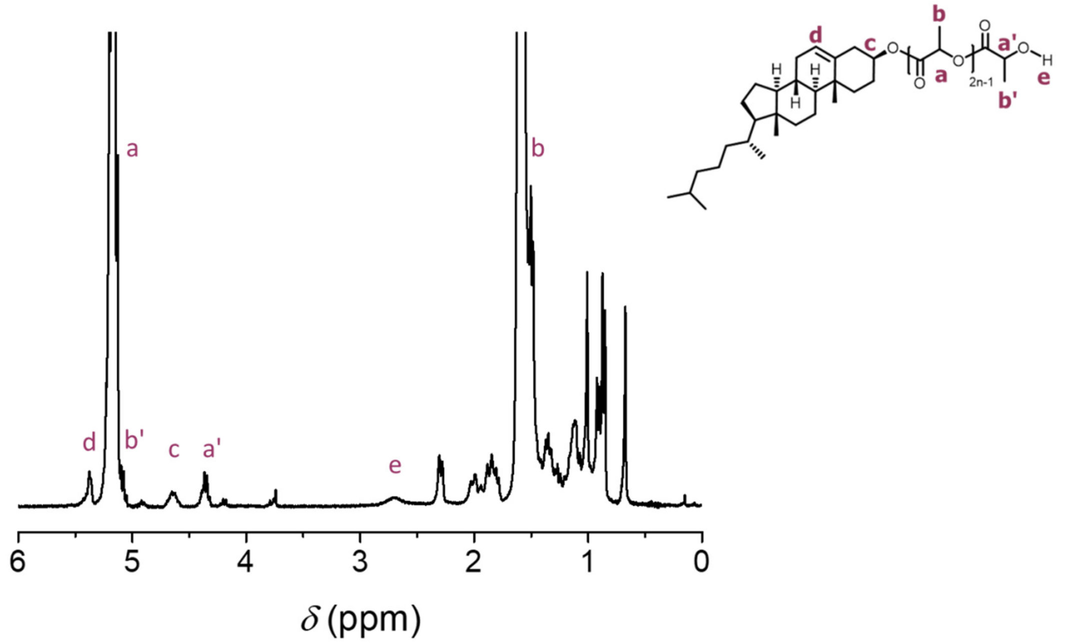

Figure S6: ^{1}H NMR spectrum (300 MHz, $CDCl_3$) of PLA-Chol-b and assignment of the signals to the schematic representation of the structure.
The peaks labeled with "a" and "d" were used to estimate the molar mass.

The final confirmation of the covalent attachment of cholesterol as an end group to PLA-Chol was provided by mass spectrometry (**Fig. S7**, **Fig. S8**). The ESI and the MALDI mass spectra revealed m/z distributions spaced by regular intervals of $\Delta m/z$ = 72, corresponding to $C_3H_4O_2$ repeating units. The fact that the full monomer lactide ($\Delta m/z = 144$) was not reflected in the mass spectra is due to the catalyst's catalytic mechanism, which inserts half a di-lactide monomer but retains the α end-group [1]. All signals in the MALDI TOF mass spectra corresponded to PLA chains initiated by cholesterol, terminated by a proton, and ionized as sodium cation adducts, also indicated by the overlay of measured and calculated isotopic patterns. It should be noted that ionization also occurred as potassium adducts during ESI TOF MS, and the corresponding doubly charged species were detected.

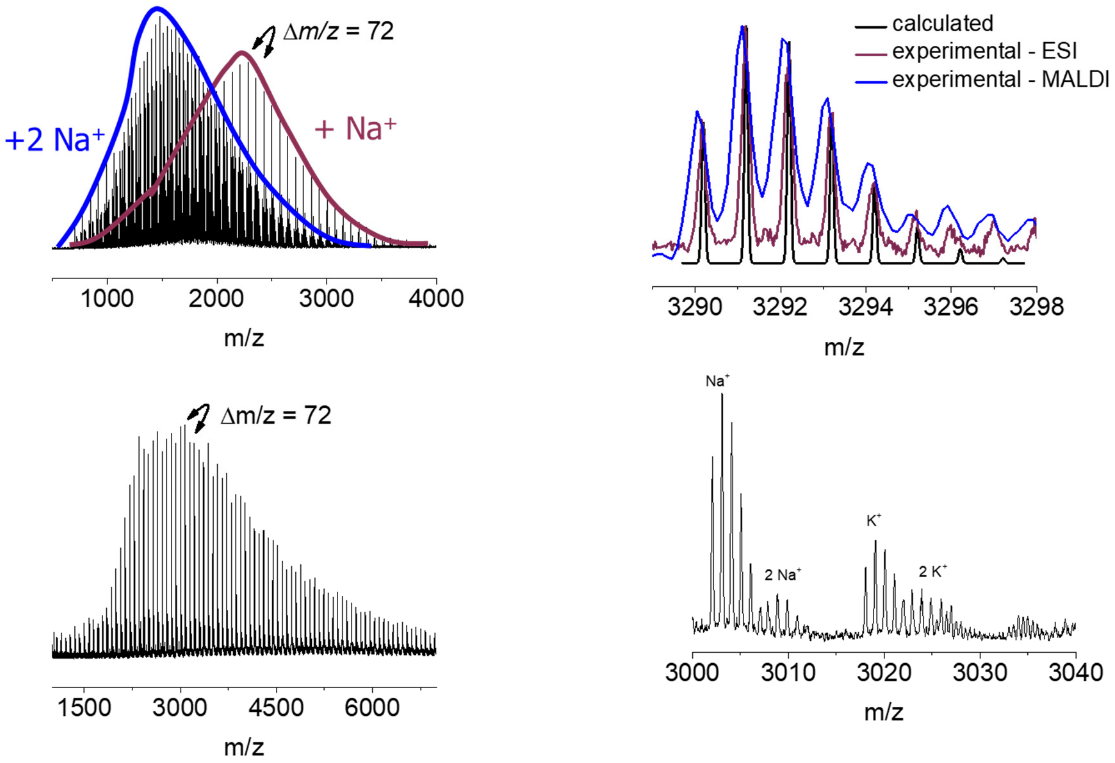

Figure S7: Characterization of PLA-Chol-a by mass spectrometry.

Top left: Full ESI TOF mass spectrum. Bottom left: Full MALDI TOF mass spectrum (DCTB, NaI). Top right: Overlay the calculated and measured isotopic patterns for the end group assignment ($C_{27}H_{45}O(C_2H_4O_2)_{40}H + Na^+$). Bottom right: Zoom into the m/z region from 3000 to 3040 of the ESI TOF mass spectrum, indicating the overlapping singly and doubly charged species due to adducts formed with sodium and potassium cations.

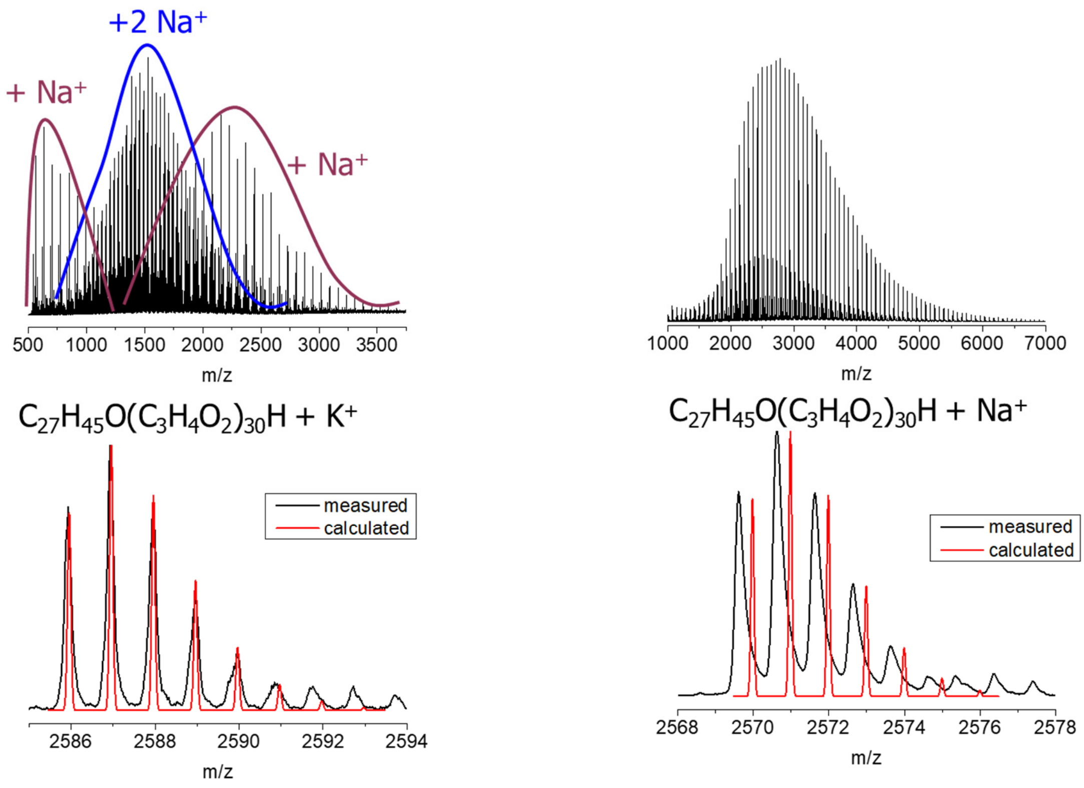

Figure S8: Characterization of PLA-Chol-b by mass spectrometry.

Top left: Full ESI TOF mass spectrum. Top right: Full MALDI TOF mass spectrum (DCTB, NaI). Bottom: Overlay of the calculated and measured isotopic patterns for end group assignment.

Materials used for the synthesis of PLA-Chol

Cholesterol and L-lactide were purchased from Sigma-Aldrich, Taufkirchen, Germany. L-lactide was purified by recrystallization from dry toluene and dried under vacuum. Tetrahydrofuran (THF) was dried by refluxing over sodium/benzophenone. The catalyst bis-(tetrahydrofuran)-calcium bis[bis(trimethylsilyl)amide] ($Ca[N(SiMe_3)_2]_2(THF)_2$) was synthesized according to previously reported procedures [3].

Instrumentation for characterization of PLA-Chol

Ring-opening polymerizations (ROP) were carried out under a nitrogen atmosphere in an MBraun UNILab glovebox workstation. ^1H nuclear magnetic resonance (NMR) spectra were recorded at room temperature in $CDCl_3$ on a Bruker Avance 300 using the residual non-deuterated solvent for chemical shift referencing. Size exclusion chromatography (SEC) measurements were performed on a Shimadzu system equipped with a CBM-20A system controller, a DGU-14A degasser, an LC-10AD vp pump, a SIL-10AD vp autosampler, and a RID-10A refractive index detector. An SDV linear guard and an SDV linear M column from PSS (Polymer Standards Service GmbH, Mainz, Germany) were used, running THF as eluent at a flow rate of 1 mL min^{-1} at 40 °C. The system was calibrated against polylactide (PLA) standards (144 to 101 000 g mol^{-1}) purchased from PSS. The matrix-assisted laser desorption ionization time-of-flight mass spectra (MALDI TOF MS) were measured on an Ultraflex III ToF / ToF instrument (Bruker Daltonics, Bremen, Germany). The instrument is equipped with an Nd-YAG laser and a collision cell. Measurements were performed in positive reflector mode using *trans*-2-[3-(4-*tert*-butyl phenyl)-2-methyl-2-pro-penylidene]malononitrile (DCTB) as matrix and sodium iodide as doping salt. The instrument was calibrated before each measurement with an external poly(methyl methacrylate) (PMMA) standard from PSS. The electrospray ionization time-of-flight mass spectra (ESI TOF MS) were acquired on a micrOTOF Q-II instrument (Bruker Daltonics) operated in the positive ion mode. The spectrometer was calibrated in the m/z range from 50 to 3000 using a calibration standard (Tunemix solution, Agilent Technologies, Waldbronn, Germany).

Experimental section for the synthesis of PLA-Chol

The ROP of L-lactide was carried out in a glove box at room temperature under a nitrogen atmosphere using THF as the solvent ($[L\text{-lactide}]_0 / [\text{cholesterol}]_0 / [Ca]_0 = 20/1/0.5$).

PLA-Chol-a: According to our previously established protocol for other bio-based alcohols [1,2], $Ca[N(SiMe_3)_2]_2(THF)_2$ (61 mg, 0.12 mmol) was dissolved in 1 mL of THF. Subsequently, this solution was added under vigorous stirring to a solution of L-lactide (0.7 g, 4.86 mmol) and cholesterol (93.9 mg, 0.24 mmol) in 3.9 mL of THF to reach an initial

monomer concentration of [L-lactide]$_0$ of 1 mol L^{-1}. After 15 min, the polymerization was quenched by adding 0.2 mL of 1 mol L^{-1} HCl solution in methanol. Analysis of an aliquot of the reaction mixture employing ^{1}H NMR spectroscopy indicated quantitative monomer conversion. Finally, **PLA-Chol-a** was isolated by precipitation in methanol and subsequent drying under reduced pressure until a constant weight was reached.

PLA-Chol-a: $M_{n,\ theo}$ = 3270 g mol^{-1}; ^{1}H NMR (300 MHz, $CDCl_3$): $M_{n,\ NMR}$ = 3500 g mol^{-1}; SEC (THF, RI detection, PLA calibration): $M_{n,\ SEC}$ = 3400 g mol^{-1}, $Đ_{SEC}$ = 1.26; MALDI TOF MS (DCTB, NaI): $M_{n,\ MALDI}$ = 4100 g mol^{-1}, $Đ_{MALDI}$ = 1.11.

PLA-Chol-b was synthesized as described above ([L-lactide]$_0$ / [cholesterol]$_0$ / [Ca]$_0$ = 20/1/0.5) using 1.4 g (9.7 mmol) of L-lactide, 188 mg (0.48 mmol) of cholesterol, 122 mg (0.24 mmol) of $Ca[N(SiMe_3)_2]_2(THF)_2$ and 9.8 mL of THF.

PLA-Chol-b: $M_{n,\ theoretical}$ = 3270 g mol^{-1}; ^{1}H NMR (300 MHz, $CDCl_3$): $M_{n,\ NMR}$ = 3000 g mol^{-1}; SEC (THF, RI detection, PLA calibration): $M_{n,\ SEC}$ = 2600 g mol^{-1}, $Đ_{SEC}$ = 1.17; MALDI TOF MS (DCTB, NaI): $M_{n,\ MALDI}$ = 3300 g mol^{-1}, $Đ_{MALDI}$ = 1.08.

References

1. Yildirim I, Crotty S, Loh CH, Festag G, Weber C, Caponi P-F, Gottschaldt M, Westerhausen M and Schubert US. *J. Polym. Sci. Part A Polym. Chem.* 2016; **54**: 437-448.
2. Yildirim I, Yildirim T, Kalden D, Festag G, Fritz N, Weber C, Schubert S, Westerhausen M and Schubert US. *Polym. Chem.* 2017; **8**: 4378-4387.
3. Zhiyuan Zhong, Pieter J. Dijkstra, Christin Birg, Matthias Westerhausen and Jan Feijen*. *Macromolecules* 2001; **34**: 3863-3868.
